# The online Tabloid Proteome: an annotated database of protein associations

**DOI:** 10.1093/nar/gkx930

**Published:** 2017-10-13

**Authors:** Surya Gupta, Demet Turan, Jan Tavernier, Lennart Martens

**Affiliations:** VIB-UGent Center for Medical Biotechnology, VIB, Ghent 9000, Belgium; Department of Biochemistry, Ghent University, Ghent 9000, Belgium; Bioinformatics Institute Ghent, Ghent University, Ghent 9000, Belgium

## Abstract

A complete knowledge of the proteome can only be attained by determining the associations between proteins, along with the nature of these associations (e.g. physical contact in protein–protein interactions, participation in complex formation or different roles in the same pathway). Despite extensive efforts in elucidating direct protein interactions, our knowledge on the complete spectrum of protein associations remains limited. We therefore developed a new approach that detects protein associations from identifications obtained after re-processing of large-scale, public mass spectrometry-based proteomics data. Our approach infers protein association based on the co-occurrence of proteins across many different proteomics experiments, and provides information that is almost completely complementary to traditional direct protein interaction studies. We here present a web interface to query and explore the associations derived from this method, called the online Tabloid Proteome. The online Tabloid Proteome also integrates biological knowledge from several existing resources to annotate our derived protein associations. The online Tabloid Proteome is freely available through a user-friendly web interface, which provides intuitive navigation and data exploration options for the user at http://iomics.ugent.be/tabloidproteome.

## INTRODUCTION

Protein–protein interactions are well known to play key roles in living cells (1,2). These protein interactions can be studied through a variety of approaches, including circuit-completion assays such as yeast-two-hybrid and MAPPIT (3), affinity purification and cross-linking mass spectrometry analyses (4) and computational analyses of protein structure (5). Yet despite the immense popularity and remarkable successes of these methods, these remain focused on direct protein interactions through actual contact between the molecules, a focus that is also found in the often complex algorithms that re-use such specialized data to determine direct protein interactions (6). As such, these approaches do not currently detect other types of biologically meaningful protein associations, for instance between proteins that are active in the same pathway. Such indirect associations are, however, likely to be as relevant as direct protein interactions for the understanding of cells and tissues in health and disease (7). Indeed, the disruption of signaling or biochemical pathways through small molecule toxins, pathogen-produced macromolecules, or cancer mutations can have profound health consequences (8). We have therefore developed a novel approach to detect novel and biologically associated protein pairs, which is based on the orthogonal re-use of publicly available data (9).

In this approach, we have used publicly available mass spectrometry-based human proteomics experiments from the PRIDE database (10). These experiments are re-processed using a pipeline build from our pride-asap (11), SearchGUI (12) and PeptideShaker (13) tools, integrated through our Pladipus (14) platform. Co-occurring protein pairs across many experiments are detected using a Jaccard Similarity metric based on the number of distinct peptides identified for each protein. Validation of the detected protein associations was performed by cross-referencing the obtained protein pairs with biological knowledge from various existing resources: pathways from Reactome (15), protein–protein interactions from IntAct (16) and BioGRID (17), protein complexes from CORUM (18), and paralog information from Ensembl (19). The majority of detected protein associations also had strong existing biological annotations, and very significantly more so than random, showing that the approach was sound (9). Further details on methodology and validation are provided in Supplementary Information S1.

Interestingly, however, only very few of the obtained associations are explained through direct protein interactions (<3% of obtained associations are known as protein interactions), which indicates that our method is very complementary to traditional protein-protein interaction analyses. Moreover, protein associations with little or no annotation were also observed, typically because one or both proteins in the pair are very poorly annotated in existing databases.

To make the results of our analysis easily available to the research community, we here present the online Tabloid Proteome as a convenient and user-friendly means to access and query all obtained protein associations, along with extensive annotation based on existing knowledgeb bases (http://iomics.ugent.be/tabloidproteome).

## DATA COLLECTION AND INTEGRATION

### Data sources

**Figure 1.**
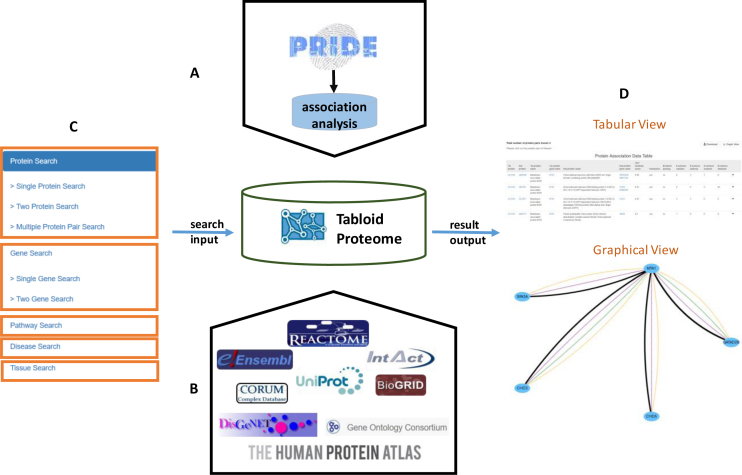
Overall view of the online Tabloid Proteome. (**A**) Raw data are derived from PRIDE database, which is further reprocessed and analyzed with a Jaccard similarity score. (**B**) The derived protein association pairs are then annotated with data from nine different resources. (**C**) The online Tabloid Proteome interface allows searches by proteins, genes, pathway, tissue and disease. (**D**) The results are depicted in two forms: a tabular view and an interactive graphical view.

Mass spectrometry-based human proteomics experiments were retrieved from the PRIDE database (release May_2015). The biological annotation included in online Tabloid Proteome is collected from six resources (Figure 1A): biological pathways are derived from Reactome (15) (V56); binary protein–protein interaction data were obtained from IntAct (16) (release 2016_01) and BioGRID (17) (version 3.4.145); protein complexes were obtained from CORUM (18) (release 2012_02), and paralog information was obtained from Ensembl (19) (version 83). To annotate protein pairs with little or no biological information, Gene Ontology (GO) annotation from UniProtKB/Swiss-Prot (20) (release 2016_06) was obtained for biological process, molecular function and cellular component annotation. Disease related information was obtained from DisGeNET (21) (version 4.0), and tissue annotation was added from The Human Protein Atlas (22) (version 17). We have used the UniProt and DAVID (23) accession number conversion.

### Database architecture and database content

All data underlying the Tabloid Proteome are stored in the Neo4j graph database (https://neo4j.com). The Tabloid Proteome currently stores 551 420 distinct associations for 4562 unique proteins pairs, all with a minimum Jaccard similarity of 0.1. These associations map against 1231 Reactome leaf pathways, 1104 CORUM complexes, 13 593 protein–protein interactions from BioGRID and IntAct, 53 tissue annotations from The Human Protein Atlas and 9697 DisGeNET diseases. These associations are currently derived from 99 distinct PRIDE projects comprising a total of 1063 assays.

### Web development

The web interface was developed using the Java Server Faces (JSF) framework, with JQuery (https://jquery.com), Bootstrap (http://getbootstrap.com) and Primefaces (https://www.primefaces.org). Visualization of the protein association graphs is performed by the Cytoscape JavaScript library (24).

## TABLOID PROTEOME FEATURES AND APPLICATION

### User interface

The online Tabloid Proteome can be queried by protein, gene, pathway, tissue or disease (Figure 1C), and results can be filtered by a Jaccard Similarity threshold score. The user can search by an entity’s name or its accession number (UniProt accession, Entrez gene ID, Reactome accession or DisGeNET ID). It is also possible to search for two or more proteins simultaneously, and for up to two genes simultaneously.

**Figure 2.**
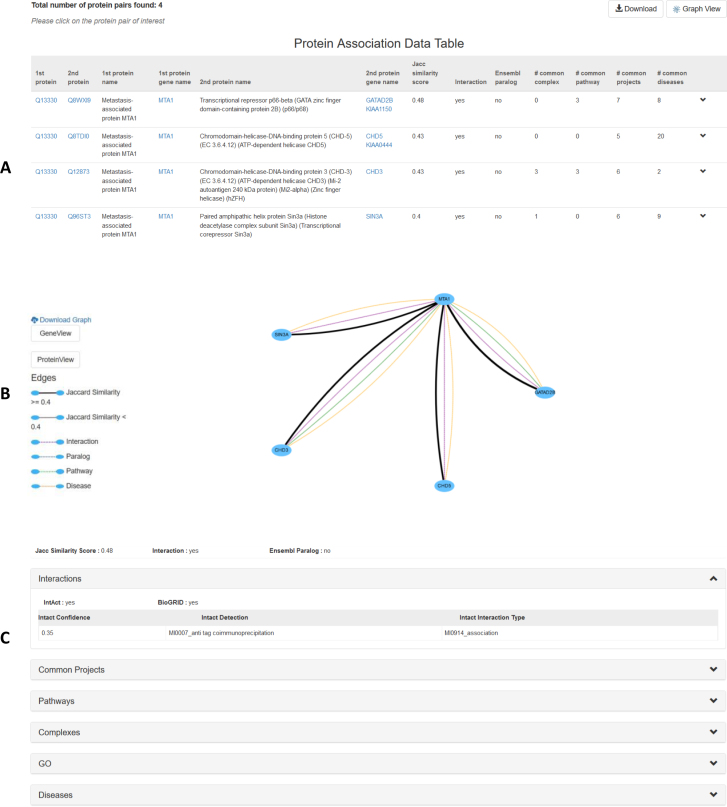
Results in the online Tabloid Proteome are displayed in two different views: a tabular and a graphical view. (**A**) In the tabular view, each row provides an overall summary of the association, while a dropdown section per row provides further information (as shown in (**C**)). (**B**) The interactive graphical view shows proteins as nodes and associations as color-coded edges. (C) Detailed information about each type of association is revealed using dropdowns the tabular view or by clicking the corresponding edge in the graphical view.

The web interface displays the results in a data table (Figure 2A). Initially, the table shows summary information in each row, which the user can then expand to see interactions, common PRIDE projects, pathways, complexes, gene ontology terms and common diseases between the two proteins in that pair (Figure 2C). Furthermore, results can also be visualized through a graph view. The user can choose to see genes or proteins, which will be rendered as nodes, while different types of associations are shown as color-coded edges (Figure 2B). All retrieved data can also be downloaded in tab-delimited or CSV format (for the tabular view), or as a PNG image (for the graph view).

A RESTful Web Service is also provided for other applications to access the Tabloid Proteome. The JSON structure and detailed information about the web service is available in the API section of the Tabloid Proteome website (http://iomics.ugent.be/tabloidproteome/tabloidApi.html).

### Example use cases

The online Tabloid Proteome can be queried in six different ways, each of which supports different use cases. These five use cases, along with some examples, are detailed below.

The first way to access the online Tabloid Proteome is via a single protein or gene search. In this case, the user provides either a single protein (e.g. Hemopexin) or gene name (e.g. *HPX*), or a single UniProt accession number (e.g. P02790) or Entrez gene ID (e.g. 3263) in the corresponding text field. When a protein or gene name, or an Entrez gene ID is used for the query, a dialog will pop up to show the matching proteins for confirmation and/or disambiguation (e.g. searching MTA1 provides two possible matching proteins). Clicking the magnifying glass icon in front of the desired protein reveals all known association partners for that protein.

Based on the query protein or gene, the resulting set of associations can consist of already well-known associations, as-yet unknown associations, or a combination of these. When querying the online Tabloid Proteome with protein MTA1 (Q13330), for instance, four associated proteins are found, all four of which are already well known to directly interact with MTA1. On the other hand, querying by protein Hemopexin (P02790), yields seven associated proteins, none of which have any prior knowledge linking it to Hemopexin. The system does indicate that many of these have a shared role in diseases. A literature search for the first associated protein, Vitamin-D binding protein (P02774, *GC*) with a Jaccard similarity score of 0.58, revealed that it has been found to be co-overexpressed with Hemopexin in breast tumor (25), which illustrates that despite a lack of existing knowledge, the association is likely biologically meaningful.

The user is thus able to consider all retrieved associations, to choose only well-known associations, or to focus exclusively on novel associations; the online Tabloid Proteome provides ample information to make such a triage easily possible.

A second use case is to focus on a protein or gene pair, by using the two protein (or two gene) search option. The supported input types are identical to the single protein or gene search. Also, as for the single search, when using anything but a UniProt accession number, a dialog pops up listing all possible protein associations for confirmation and/or disambiguation.

Importantly, for a two protein query, the online Tabloid Proteome also automatically retrieves indirect associations if the searched pair does not have a direct association. For instance, when queried with the protein accession numbers Q96ST3 and Q8TDI0, no direct associations are found but the system presents the user with a possible indirect relation between both proteins, as both are associated to *MTA1*.

A third use case is to query the online Tabloid Proteome for multiple protein association pairs, which is supported by the multiple pair protein search option. Here, the user can upload a file with protein pairs, which are then queried. Interestingly, the multiple pair protein search also allows users to provide their own links for these pairs in the uploaded file, which are then visualized in the graphical view alongside the other edges.

The online Tabloid Proteome search is not limited to only protein or gene-based searches.

Indeed, the fourth use case allows the system to be queried by a Reactome pathway, using either a pathway name or Reactome accession number. The input text can be generic, such as ‘metabolism’, or can be specific, for instance ‘Pyruvate metabolism’ or Reactome accession number ‘R-HSA-70268’. The result is a list of all known associated protein pairs in which both members are involved in the provided pathway. Each individual pair can then be explored in more detail by clicking the magnifying glass icon for that pair in the overview table.

The fifth use case then, consists of retrieving all associated protein pairs that are detected for a particular disease. This can be achieved by querying the online Tabloid Proteome with either a disease name or a DisGeNET ID. As for the pathways, the query can be generic (e.g. ‘cancer’, which will result in 139 matched diseases), or selective (e.g. ‘Cancer of Nasopharynx’ or DisGeNET ID: ‘C0238301’).

The sixth case allows the user to restrict their search for associated protein pairs by tissue annotation, as provided by The Human Protein Atlas. This search can be performed by selecting one of the 53 tissue names, for example, ‘lung’, resulting in 653 protein pairs. Similar to the disease and pathway search, the results are shown in an overview table.

## DISCUSSION AND OUTLOOK

The online Tabloid Proteome is an easily searchable website that presents protein associations derived from public mass spectrometry-based proteomics datasets, along with annotation of these associations using information from a variety of existing knowledge bases. Due to the strong complementarity with existing approaches (most notably with direct protein-protein interaction studies) the online Tabloid Proteome provides unique added value for researchers that are interested in understanding the relations between proteins.

Future plans for the online Tabloid Proteome focus on the inclusion of association data obtained from public proteomics data from other model organisms (notably mouse, yeast and Arabidopsis). This will also allow orthologous association pairs that have been conserved across evolution to be derived. Additionally, we also compared the protein pairs to information from the STRING database (26) and found 296 protein pairs that had text mining and/or co-evolution annotation in STRING, but that had no annotation in any of the other annotation databases. We will therefore also add STRING database annotations to the online Tabloid Proteome.

## AVAILABILITY

The online Tabloid Proteome is freely available via http://iomics.ugent.be/tabloidproteome. The documentation for database usage is available via http://genesis.ugent.be/uvpublicdata/Tabloid_Proteome/TabloidProteome1.2_documentation.pdf and the documentation for API REST is available via http://iomics.ugent.be/tabloidproteome/tabloidApi.html.

## Supplementary Material

Supplementary Data
