# Supplementary material for: The online Tabloid Proteome: an annotated database of protein associations
Source: Nucleic Acids Res. 2017 Oct 13;46(Database issue):D581–5. doi: 10.1093/nar/gkx930 (PMC5753264; doi:10.1093/nar/gkx930)
Supplement: Supplementary Data [file gkx930_supp.pdf]

S1 methodology and validation

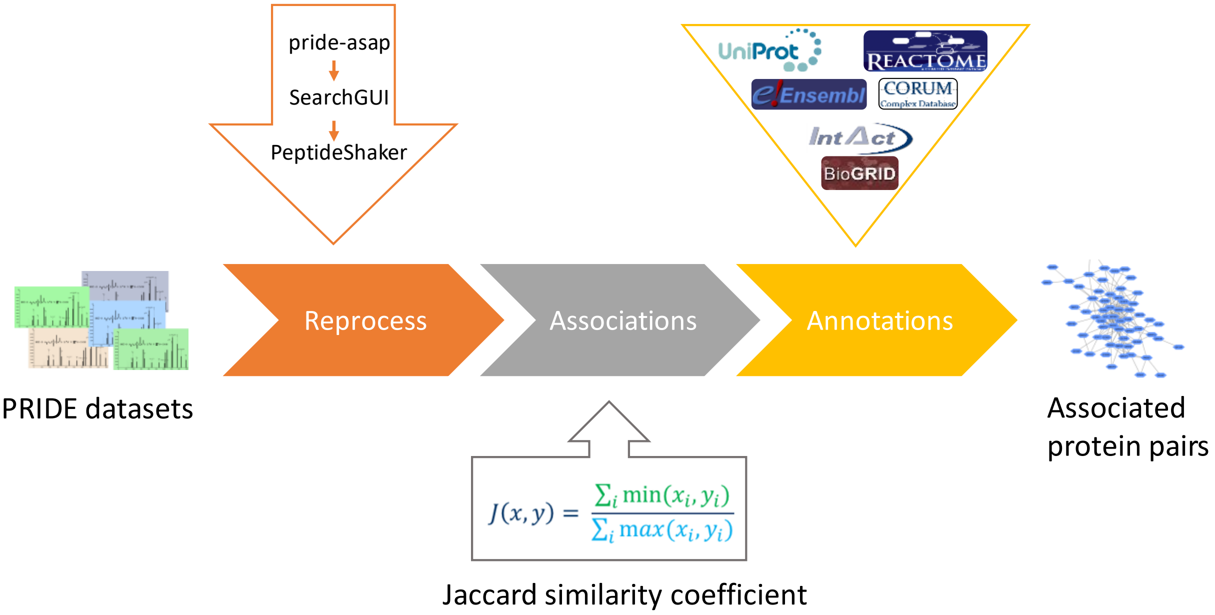

Figure S1. An outline of the workflow to generate and annotate associated protein pairs from mass spectrometry based proteomics studies, derived from PRIDE.

Complete mass-spectrometry based human proteomics projects was obtained from the PRIDE database (downloaded in MAY 2015) using PRIDE webservice. Here, complete projects refer to the projects which contains both identification and fragmentation mass spectra. The analysis of the derived data is performed in three major steps: reprocess, associations and annotations (as shown in Figure S1).

Reprocessing is performed using three existing tool: pride-asap, SearchGUI and PeptideShaker. Pride-asap uses available identification for each dataset, to infers optimal search parameters (such as precursor ion and fragment ion mass tolerances, most relevant variable and fixed modifications and choice of digestion enzyme), for each experiment. These search parameter settings from pride-asap, were further used by SearchGUI and PeptideShaker, for reprocessing. In SearchGUI, three search engines: MS-GF+ (1), MyriMatch (2) and X!tandem (3), was used for sequence database search, against human proteome complement of UniProt Swiss-Prot (only canonical sequence). Furthermore, common Repository of Adventitious Proteins (cRAP) from The Global Proteome Machine (GPM) (4) database was used in the sequence database to address all commonly found contaminant proteins. PeptideShaker process and integrate the search results from the three search engines, and control local FDR at PSM level.

Protein inferences derived from PeptideShaker, was further filtered for only UniProt-SwissProt identified proteins. Furthermore, distinct peptide count for each protein in each experiment was further used to

detect co-occurring proteins. The weight of protein co-occurrence across experiments is calculated, using statistical Jaccard similarity coefficient (as shown in Figure S1).

Protein pairs with a jaccard similarity coefficient of 0.4 were mapped to five knowledgebases, to assign potential biological relevance. Reactome was used to annotate co-occurring proteins with same biological pathways. Ensembl was used to detect paralogs. IntAct, and BioGRID was used for detecting protein-protein interaction and CORUM database was used to detect co-occurring proteins, found to form same protein-complexes. Furthermore, Gene Ontology (GO) biological process, molecular function and cellular component was used to extend the biological annotation for the unannotated protein pairs.

Of the 2325 protein pairs that pass the Jaccard similarity threshold, we have successfully been able to map 81% of pairs. 68% protein pairs were mapped with existing biological knowledgebases; Reactome, IntAct, BioGRID, CORUM, and Ensembl, and 13% with possible biological connection using Gene Ontology (GO) terms (5).

To verify the fundamental validity of our approach, we compared the level of biological association for the original results with that of protein pairs from randomized associations. When we mapped 2325 randomly selected pairs to the knowledgebases, we only found 40 annotated protein pairs (on average), and 69 annotated protein pairs (at maximum) over the 1000 iterations. As a result, the difference between real and random data is extremely significant. For few unannotated protein pairs, with high Jaccard similarity score, we have also performed manual validation using literature search and text-mining annotation from String database.

1. Kim,S. and Pevzner,P.A. (2014) MS-GF+ makes progress towards a universal database search tool for proteomics. *Nat. Commun.*, **5**, 5277.

2. Tabb,D.L., Fernando,C.G. and Chambers,M.C. (2007) MyriMatch: highly accurate tandem mass spectral peptide identification by multivariate hypergeometric analysis. *J. Proteome Res.*, **6**, 654–61.

3. Fenyö,D. and Beavis,R.C. (2003) A Method for Assessing the Statistical Significance of Mass Spectrometry-Based Protein Identifications Using General Scoring Schemes. *Anal. Chem.*, **75**, 768–774.

4. Robertson Craig,†, John P. Cortens,‡ and and Ronald C. Beavis*, †,‡ (2004) Open Source System for Analyzing, Validating, and Storing Protein Identification Data. 10.1021/PR049882H.

5. Gupta,S., Verheggen,K., Tavernier,J. and Martens,L. (2017) Unbiased Protein Association Study on the Public Human Proteome Reveals Biological Connections between Co-Occurring Protein Pairs. *J. Proteome Res.*, **16**, 2204–2212.
